# Risk of Hematologic Malignant Neoplasms after Postoperative Treatment of Breast Cancer

**DOI:** 10.3390/cancers11101463

**Published:** 2019-09-29

**Authors:** Marie Joelle Jabagi, Anthony Goncalves, Norbert Vey, Thien Le Tri, Mahmoud Zureik, Rosemary Dray-Spira

**Affiliations:** 1 EPI-PHARE (French National Agency for Medicines and Health Products Safety (ANSM) and French National Health Insurance (CNAM)), 143 Boulevard Anatole, 93200 Saint-Denis, France; thien.le-tri@ansm.sante.fr (T.L.T.); mahmoud.zureik@ansm.sante.fr (M.Z.); rosemary.dray-spira@ansm.sante.fr (R.D.-S.); 2 Aix-Marseille Univ, Inserm, CNRS, Institut Paoli-Calmettes, CRCM, 13009 Marseille, France; goncalvesa@ipc.unicancer.fr (A.G.); veyn@ipc.unicancer.fr (N.V.); 3 Versailles Saint-Quentin-en-Yvelines University, 78000 Versailles, France

**Keywords:** Breast Cancer, Secondary Hematologic Malignant Neoplasms, Secondary Cancers, Leukemia, Lymphoma, Treatment-related Complications, Chemotherapy, Radiotherapy, Hormonal therapy, Epidemiology

## Abstract

An indirect consequence of the improved long-term survival seen in patients with breast cancer (BC) is the increased risk of hematologic malignant neoplasms (HM). This study aimed to analyze the role of postoperative treatment for BC in the development of subsequent HM. Using the French National Health Data System, we examined the HM risks in patients diagnosed with an incident primary breast cancer between 2007 and 2015, who underwent surgery as first-line treatment for BC. Main outcomes were acute myeloid leukemia (AML), Myelodysplastic syndrome (MDS), myeloproliferative neoplasms (MPNs), multiple myeloma (MM), Hodgkin’s lymphoma or non-Hodgkin’s lymphoma (HL/NHL), and acute lymphoblastic leukemia or lymphocytic lymphoma (ALL/LL). Analyses were censored at HM occurrence, death, loss to follow up, or December 2017. The risk of each type of HM was compared according to the initial postoperative treatment of breast cancer. Of a total of 324,056 BC survivors, 15.5% underwent surgery only, 46.7% received radiotherapy after surgery, 4.3% received chemotherapy after surgery, and 33.5% received all three modalities. Overall, 2236 cases of hematologic malignancies occurred. Compared to the surgery alone group, AML was significantly increased after surgery plus radiation (aHR, 1.5; 95% CI, 1.0–2.1), surgery plus chemotherapy (aHR, 2.1; 95% CI, 1.2–3.6) and all modalities (aHR, 3.3; 95% CI, 2.3–4.7). MDS was significantly increased after surgery plus chemotherapy (aHR, 1.7; 95% CI, 1.1–2.5) or after all modalities (aHR, 1.4; 95% CI, 1.1–1.8). HL/NHL were significantly increased only in the radiotherapy and surgery group (aHR, 1.3; 95% CI, 1.0–1.6). A nonsignificant increase of ALL/LL (aHR, 1.8; 95% CI, 0.6–3.5) was noted after chemotherapy and with all three modalities (aHR, 1.4; 95% CI, 0.7–2.8). Our population based study revealed increased risks of various HM associated with postoperative BC treatment. The added benefit of chemotherapy and radiation therapy should take into consideration these long-term complications.

## 1. Introduction

Early detection and improved therapeutic options including, endocrine therapy, cytotoxic chemotherapy, targeted therapies, and radiation therapy have led to improved survival in breast cancer (BC), the most common and often curable malignant solid tumor in women. A major concern, however, is the rising rates of secondary malignancies in this steadily growing population of cancer survivors. 

Myeloid neoplasms such as acute myeloid leukemia (AML) and Myelodysplastic syndrome (MDS) could be long-term complications of chemotherapy [1,2,3] and radiation therapy [4,5]. Breast cancer has ranked among the most common malignant solid tumors at risk for their development [6,7]. Nevertheless, the exact magnitude of the risk of these two secondary hematological malignancies after a postoperative treatment for breast cancer in the current context is not clear. Moreover, previous studies based on hospital series have reported treatment-related lymphocytic leukemia and lymphomas [8,9,10,11,12]. However, the risk for developing these hematological malignancies (HM) after first-line BC treatment remains an unexplored question. 

In a recent study [13], we used the French National Health Data System to estimate the real-life incidence of various hematologic malignancies in BC survivors in the recent era (2007–2015). Herein, we examined the same national cohort to quantify the risk of secondary hematologic malignancies in BC patients according to the specific treatment modalities delivered in the postoperative setting. This robust population-based cohort study allowed us to have the sample size and the follow up needed to thoroughly investigate these therapy-related complications. 

## 2. Materials and Methods 

### 2.1. Data Source

We used data from the French National Health Data System (SNDS). The SNDS database includes all French residents’ health expenses. In France, the General Insurance Plan extends to all private and public sector employees and students, covering approximately 88% of the French population. 

The SNDS includes individual information on sociodemographic characteristics, the cause of death and all patients’ health reimbursements including outpatient medical care, laboratory tests, dispensed drugs, and medical details (including any severe or costly medical condition). An anonymous, unique subject identifier links information from the database to the national hospital and discharge database which covers all public and private hospitals admissions and discharges. It contains data on the diagnoses (coded in the International Statistical Classification of Diseases and Related Health Problems, Tenth Revision (ICD-10)) and type of surgical acts and treatments provided during hospital stays. Various epidemiologic studies have used these databases [14,15].

The French Data Protection Supervisory Authority approved this study. 

### 2.2. Study Population

Patients eligible for this study were women 20 to 85 years old, not suffering from dementia, with incident primary breast cancer (ICD-10 codes; C50: Invasive Breast Cancer) diagnosed between 2007 and 2015 and registered in the general health insurance coverage program. To restrict the study to incident cases of primary BC, we excluded patients with a history of any cancer. In addition, we limited our study to non-dementia patients aged 20 to 85 years because of different treatment approaches in younger and elderly patients and a likely under-reporting of cancer in the geriatric demented population (< 85 years). This cohort has been extensively described in a previous study [13].

The main treatment for nonmetastatic invasive breast cancer being surgical resection [16], we further restricted the study population to BC patients who received a surgical intervention as their primary treatment for Breast Cancer in the six months following diagnosis. Therefore, patients with initial metastatic disease, as well as patients receiving neoadjuvant systemic treatment for locally advanced or large tumors, were excluded [17].

Patients were followed-up starting one year after the initial BC-surgery until hematologic malignancy diagnosis, death, loss to follow up, or end of the study (December 31, 2017), whichever came first. Patients who developed secondary malignancies or died during the first year were hence not included. 

### 2.3. Exposure Definition 

Surgery in the six months following breast cancer diagnosis, as defined using the common classification of medical procedure codes (CCAM), was considered the date of inclusion (Table S1 in supplement). The main exposure considered as the initial postsurgical treatment for BC was defined during the first year following inclusion as follows; Exposure to chemotherapy in the 6 months following surgery for BC, radiation therapy and hormonal therapy in the year following BC-surgery. Chemotherapy for BC is administered at hospitals and radiation therapy can be delivered either at hospitals or at specialized private centers. They are identified in the database using ICD-10 codes and *CCAM* (Table S1 in supplement). Hormonal therapy is dispensed by pharmacies and is identified using anatomical therapeutic chemical classification system codes (ATC) (Table S1 in supplement). 

### 2.4. Covariates

Sociodemographic characteristics included age, type of medical care structure, and affiliation to complementary universal health insurance. Universal health insurance offers full free healthcare to people with low income. Lifestyle habits included alcohol use disorder, smoking-related conditions, and obesity. Medical history defined at cohort entry included hypertension, diabetes, hyperlipidemia, heart disease, HIV and hepatitis B and C. Comedications were identified with prescriptions reimbursed at least once in the six months preceding cohort inclusion. Detailed definitions of covariates are described in Table S2 in the supplement. 

### 2.5. Outcomes 

The main outcomes were incident hematologic malignant neoplasm cases occurring at least one year after breast cancer surgery and were identified using ICD-10 codes. Hematologic malignant neoplasms were divided into myeloid and lymphoid neoplasms. Myeloid neoplasm included acute myeloid leukemia (AML), myelodysplastic syndrome (MDS), and myeloproliferative neoplasms (MPNs). Lymphoid neoplasms included multiple myeloma (MM), Hodgkin’s or non-Hodgkin’s lymphoma (HL/NHL), and Acute Lymphoblastic Leukemia or Lymphocytic Lymphoma (ALL/LL). Detailed definitions of the outcomes are presented in Table S3 in the supplement. 

### 2.6. Statistical Analyses 

The risk of each hematologic malignancy subtype was compared between the four mutually exclusive groups (surgery alone, surgery with radiotherapy, surgery with chemotherapy, and all three modalities), considering the surgery alone group as the control group. Crude incidence rates per 100,000 person-years at risk, and their corresponding 95% confidence intervals (CIs) [18], were calculated for each type of hematologic malignancy for the whole cohort and by treatment modality group. Hazard ratios, and their 95% Cis, were obtained from multivariate Cox proportional hazard models adjusted for age at inclusion, affiliation to complementary universal health Insurance, year of inclusion, type of structure, hormone therapy, severe alcoholism, heavy smoking, morbid obesity, immunosuppressant, hepatitis B and C, and HIV. In a complementary adjusted multivariate Cox analysis, we considered chemotherapy and radiotherapy as single groups of exposure and we studied their effect on hematologic malignancies’ risk. Their interaction with age were also investigated. Overall survival post-HM diagnosis was calculated for each type of HM as the time from HM diagnosis till death, loss to follow up or December 31, 2017 using the Kaplan–Meier method. 

Sensitivity analyses were conducted using Fine and Gray’s method [19] considering patients who died before developing an HM as having a competing event. Cumulative incidence of HM comparing the four mutually exclusive groups were reported in the presence of death as competing risk. 

All analyses were performed with SAS software version 9.4 (SAS Institute Inc.)). 

## 3. Results 

### 3.1. Patient’s Characteristics 

**Figure 1:**
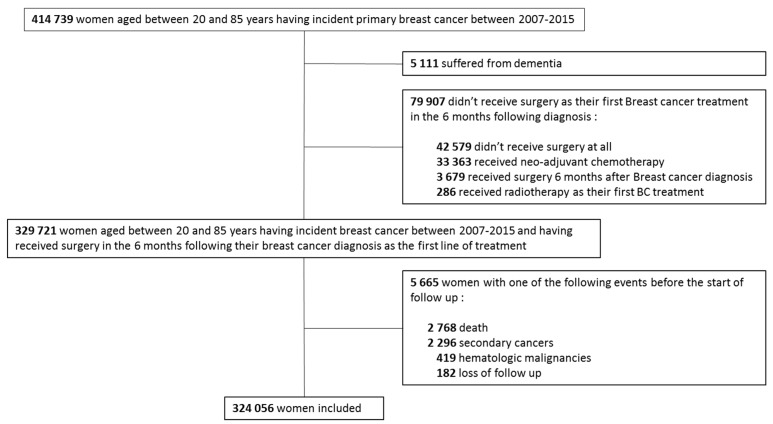
Study flowchart.

We included 324,056 women in the cohort (Figure 1) with a median follow up time of 4.95 years (interquartile range, 2.8–7.3 years) and a 10-year overall survival rate after breast cancer of 82% with 25,701 women dying during follow up.

During the first year after surgery for BC, 151,360 (46.7%) received radiotherapy only, 13,937 (4.3%) received chemotherapy only, 108,436 (33.5%) received both chemotherapy and radiotherapy, and 50,323 (15%) received neither radiotherapy nor chemotherapy. Women of our cohort had a median age at cohort entry of 59 years (Table 1). Women who received chemotherapy only (median: 56 years) or chemotherapy combined to radiotherapy (median: 55 yrs.) were younger than those who did not receive chemotherapy at all (surgery only (median: 63 yrs.) or surgery and radiotherapy (median: 60 yrs.)). Women in the four groups of exposure had very similar socioeconomic status and healthy lifestyle. However patients who received chemotherapy had less comorbidities and prior to inclusion medication consumptions. Radical mastectomy was the most frequent BC-removal type of surgery (80.8%) performed in patients treated with chemotherapy only and was very rarely performed in patients treated with radiotherapy only (6.7%). In fact, a more breast-conserving surgery such as partial mastectomy or lumpectomy (93.3%) was performed in patients treated with radiotherapy only and in the three modalities group (76.3%). Approximately 70% of patients were treated with hormone therapy, mostly aromatase inhibitors (48.3%) and selective estrogen receptor modulators (23.5%). 

### 3.2. Hematologic Malignancies’ Occurrence in the Whole Cohort and by Exposure Groups

Overall, 2236 cases of hematologic malignancies occurred (Table 2). Cases of myeloid neoplasm comprised 436 cases of AML (Incidence Rate [IR] per 100,000 person-years, 26.5; 95% CI, 24.0–29.0), 552 cases of MDS (IR, 33.5; 95% CI, 30.8–36.4), and 194 cases of MPN (IR, 11.8; 95% CI, 10.2–13.5). Lymphoid neoplasm cases included 310 cases of MM (IR, 18.8; 95% CI, 16.8–21.0), 668 cases of HL/NHL (IR, 40.6; 95% CI, 37.5–43.8), and 76 cases of ALL/LL (IR, 4.6; 95% CI, 3.6–5.8) (Table 2). Cumulative incidence function yielded similar results, showing that the exposure groups with the highest risk for each type of hematologic malignancy stayed the same over the course of the 10-years (Figure S1 in supplement). The median time from breast cancer surgery to hematologic malignancy occurrence ranged from 3.2 (2.1–5.0) years for AML to 4.7 (3.0–6.5) for HL/NHL (Table 2). Median survival after HM diagnosis ranged from 0.8 years for AML and ALL/LL to 6.7 years for HL/NHL (Figure S2 in supplement). 

### 3.3. Risk of HM According to BC Treatment Modalities 

In multivariable analysis, a significant increase in the risk of AML was observed among patients treated with surgery plus radiation (aHR, 1.5; 95% CI, 1.0–2.1; *p* = 0.04), surgery plus chemotherapy (aHR, 2.1; 95% CI, 1.2–3.6; *p* = 0.007), and all three modalities (aHR, 3.3; 95% CI, 2.3–4.7; *p* ≤ 0.0001) compared to surgery alone. MDS was significantly increased among patients treated by both surgery and chemotherapy (aHR, 1.7; 95% CI, 1.1–2.5; *p* = 0.02) and among those treated with all three modalities (aHR, 1.4; 95% CI, 1.1–1.8; *p* = 0.01). HL/NHL was significantly increased only in the radiotherapy and surgery group (aHR, 1.3; 95% CI, 1.0–1.6; *p* = 0.02) compared to the surgery alone group. A nonsignificant increase of ALL/LL (aHR, 1.8; 95% CI, 0.6–3.5; *p* = 0.3) was observed among patients treated with both surgery and chemotherapy and those treated with all three modalities (aHR, 1.4; 95% CI, 0.7–2.8; *p* = 0.4) (Table 3). Increasing age, immunosuppressant, and morbid obesity were also independently significantly associated with a higher risk of certain types of hematologic malignancies (Table S4 in supplement). In another multivariable analysis, when looking into the effect of chemotherapy vs. no chemotherapy adjusted on radiotherapy and other covariables, the risk of AML (aHR, 2.3; 95% CI, 1.8–2.8; *p* ≤ 0.0001), MDS (aHR, 1.5; 95% CI, 1.2–1.8; *p* ≤ 0.0001), and ALL/LL (aHR, 1.7; 95% CI, 1.0–2.7; *p* = 0.03) were all significantly increased (Table 4). Patients treated by radiotherapy, adjusting for chemotherapy, had a significantly higher risk of AML (aHR, 1.7; 95% CI, 1.0–2.7; *p* = 0.005) and HL/NHL (aHR, 1.2; 95% CI, 1.0–1.5; *p* = 0.05) compared to patients not treated by radiotherapy at all (Table 4). 

Risk of MDS was slightly, not significantly increased for patients treated with hormone therapy versus patients not treated with hormone therapy (aHR, 1.2; 95% CI, 1.0–1.5; *p* = 0.07). For the other hematologic malignancy types, the risk did not differ between patients exposed and unexposed to hormonal therapy (Table S5 in supplement). 

Results regarding the risks of hematologic malignancy after exposure to chemotherapy and radiotherapy remained unchanged in the sensitivity analysis taking into consideration death as a competitive event (Table S6 in supplement). 

### 3.4. Risk of Hematologic Malignancies Stratified by Age 

**Figure 2:**
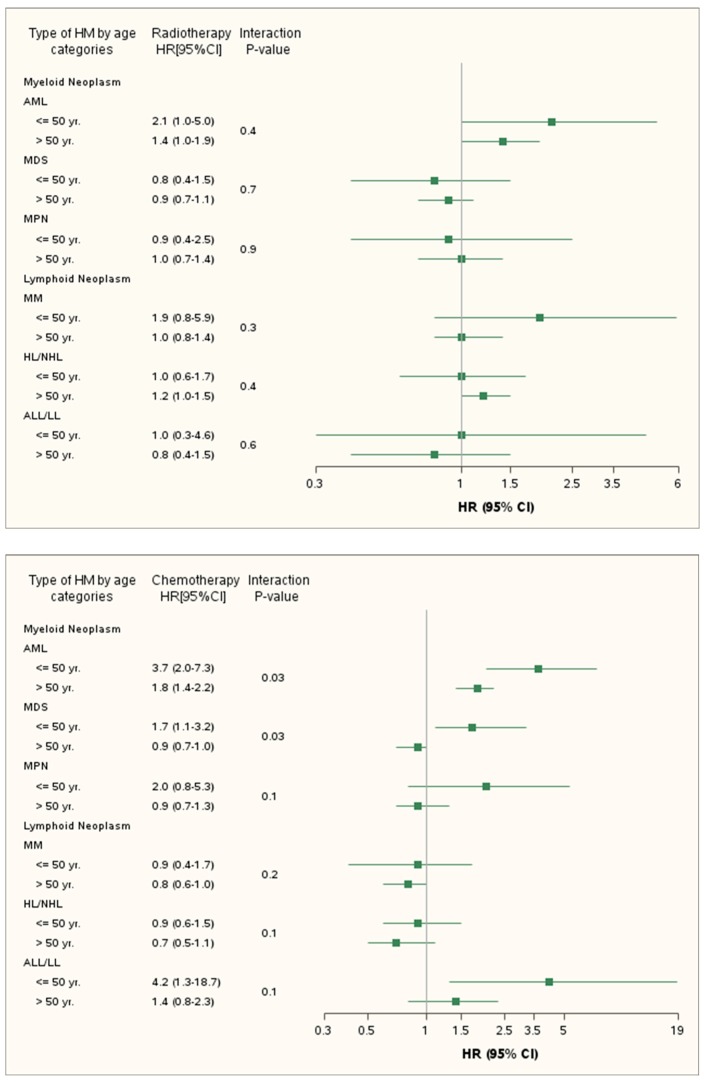
Risk of hematologic malignancies by age after chemotherapy and radiotherapy. Abbreviations: HM. Hematologic Malignancies; CI. Confidence Interval; AMl. Acute myeloid Leukemia; MDS. Myelodysplastic syndrome; MPN. Myeloproliferative Neoplasm; MM. Multiple Myeloma; HL/NHL. Hodgkin’s and Non-Hodgkin’s lymphoma; ALL/LL. Acute lymphoblastic leukemia lymphocytic lymphoma. *Chemotherapy HR: Adjusted hazard ratio in the multivariable Cox model adjusted for radiotherapy, hormonal therapy and baseline characteristics including age at inclusion. affiliation to complementary Universal health insurance. Year of inclusion. type of structure. severe alcoholism. heavy smokers. morbid obesity. immunosuppressant. hepatitis B & C. HIV. *Radiotherapy HR: Adjusted Hazard Ratio in the multivariable Cox model adjusted for chemotherapy, hormonal therapy and baseline characteristics including age at inclusion. affiliation to complementary Universal health insurance. Year of inclusion. type of structure. severe alcoholism. heavy smokers. morbid obesity. immunosuppressant. hepatitis B and C. HIV.

In multivariable analyses by age, chemotherapy seemed to be associated with a higher risk of hematologic malignancies in younger patients. However, only the risks of AML and MDS were significantly higher among patients treated at younger ages (*p*_interaction (AML)_ = *p*_interaction (MDS)_ = 0.03). In contrast, radiotherapy was not associated with a significant difference between the two age groups (Figure 2). 

## 4. Discussion 

In this population-based cohort of 324,056 breast cancer survivors with a median follow-up of 5 years, we examined the risk of the different HM subtypes related to postoperative initial BC therapy. There was a significant increase in AML and HL/NHL risk if radiotherapy was added to surgery and a marked significant increase of AML, and to a lesser extent of MDS and ALL/LL, if chemotherapy with or without radiation therapy was added to surgery. Hormonal therapy did not increase significantly any of the hematologic malignancies’ risk. Young breast cancer survivors (≤ 50 years) seemed to have a significantly higher risk of developing AML, when exposed to chemotherapy, compared to older patients. However, the risk stayed significantly high in the two age groups. For ALL/LL and MDS, only younger patients had a very pronounced increased risk after chemotherapy. 

Our real-life data showed therapeutic practices similar to what was recommended and reported by others. The standard approaches for surgical resection in BC are either radical mastectomy or partial mastectomy (lumpectomy) followed by radiation [16]. These two approaches are equivalent with regards to relapse-free survival [20]. In our cohort, the majority of patients who received radiation therapy have had a partial mastectomy or lumpectomy. Younger patients of our study were more prone to receive chemotherapy. This was expected since young women are more likely to present with a more aggressive and advanced stage BC [21]. Older women were mostly found in the surgery/radiotherapy only group. Overall, 69.5% of the women of our study received endocrine therapy for BC with the surgery/radiotherapy group having the higher proportion of hormonal therapy use. In fact, hormone receptor-positive (HR+) tumors constitute 70% of invasive breast cancers [22] and are more likely to occur in older women [23].

In our large scale study, we found a 2.2-fold increased risk of AML after chemotherapy and a 3.3-fold increased risk after chemoradiotherapy in BC survivors. This excess of AML risk already established after the use of some cytotoxic agents such as alkylating agents and topoisomerase II inhibitors [6,24,25] still persisted for patients of our study treated for BC in the modern era going from 2007 to 2015. In fact, other than the introduction of taxanes in the early 2000s [16], the chemotherapy regimens for BC did not change drastically during the last two decades. Cyclophosphamide and anthracyclines are still widely used and the exposure to taxanes is not known to be associated with therapy-related hematologic malignancies [26]. Radiotherapy was also responsible for a 1.5-fold increase in the AML risk which explains the fact that AML risk was higher after chemo-radiotherapy compared to chemotherapy alone. Although, radiation exposure after breast cancer has been linked to leukemia [5,27] the effect of radiation is not well characterized as most studies did not include radiation treatment patients as a separate group. 

An excess risk for MDS of 1.5-fold was found after chemotherapy, whereas radiation therapy did not appear to be associated with an increased risk in BC survivors of our study. Several studies looked at AML/MDS as a sole entity and found that chemotherapy and radiotherapy increased the risk [1,2,28]. However, no studies reported the association of previous BC treatment on the risk of MDS as a separate group. 

A unique feature of our study, was the ability to quantify risks of ALL/LL after chemotherapy for breast cancer in the current treatment era. We found a 1.7-fold increased risk of ALL/LL after chemotherapy while exposure to radiation therapy did not seem to be associated with an increased risk. Given the scarce frequency of ALL, most literature reported secondary ALL as case reports or case series [11,12]. Other studies could not confirm nor exclude a relationship between the use of chemotherapy and ALL/LL occurrence [2,29] due to the low number of cases in their datasets. Yet, some cytogenetics studies have shown that the characteristics of secondary ALL are parallel to the well-recognized cytogenetic alterations found in secondary AML [10,11,30]. Lymphomas had a small but significant 1.3-fold increase after radiation therapy. There is weak but consistent evidence linking radiotherapy exposure to lymphomas in solid cancer survivors [31,32]. Even if the association between radiation exposure and lymphomas is recognized in the therapeutic settings, our results provide what we believe to be the first clear evidence of the excess lymphomas risk following radiotherapy for breast cancer. 

An intriguing, and clinically relevant, finding of our study was that age was an important modifier of AML, MDS, and ALL/LL risk after chemotherapy. The administration of chemotherapy for BC diagnosed in patients aged 50 years and younger poses the highest risk for AML. The risk remaining significantly high in the two age groups. Other studies have already suggested an increased risk of subsequent diagnosis of AML in younger breast cancer survivors and a decreasing risk with increasing age [1,3,4,33]. For ALL/LL and MDS though, only younger patients seemed to be at risk after chemotherapy in our study. Genetic predisposition and intrinsic susceptibility to leukemogenic exposures, in addition to the fact that younger patients suffering from a poorer prognostic BC are treated with more intensive chemotherapy, represent a plausible explanation to this age-related susceptibility. In regards to radiation therapy, younger BC survivors did not seem to be more susceptible to secondary hematologic malignancies in our study, even though some reported an increased sensitivity to leukemia induction in younger women [34,35].

Latency time between breast cancer and leukemia (AML and ALL/LL) was the shortest in patients exposed to chemotherapy compared to surgery only. In fact, treatment-related AML has been reported within the first several years after cytotoxic chemotherapy for BC [4,5]. The median interval time of 2.7 to 2.8 years between BC diagnosis and the onset of AML in patients receiving chemotherapy is very much comparable to other studies having the same follow-up time length as ours [36]. Time proximity from primary BC could be an indicator of treatment-related disease. 

Hematologic malignancies in our BC survivors were associated with poor survival as reported by other studies [24,37,38]. Survival for AML and ALL/LL in our study was measured in months, not years, making these leukemias the worst prognosis cancers. Accumulation of chromosomal aberrations and mutations from prior chemotherapies are thought to be the cause of the aggressive and treatment-resistant behavior [39,40,41].

One of the study’s limitations was the lack of data on chemotherapy agents and their actual doses. However, the main adjuvant chemotherapy regimen for breast cancer is based on cyclophosphamide and anthracyclines with some patients additionally receiving taxane [16]. Cyclophosphamide and anthracyclines, are known to be associated with 2 different types of acute myeloid leukemia (AML) [6,24,25], whereas exposure to taxanes is not known to be associated with therapy-related hematologic malignancies [26]. Further investigations will be needed to elucidate the role that chemotherapy and cumulative therapies play in the development of ALL/LL. Further investigations will also be needed to elucidate the role of cumulative therapies and to better understand differences in risk by age at exposure based on treatment duration and intensity. Cytogenetic information and family history could have also helped us identify genetic predisposition and host susceptibility.

Despite these limitations, our study is based on a nationwide population-based data with a relatively long-term follow up in the recent therapeutic era. We obtained complete real-life data on each patient with very homogeneous treatment strategies. We believe that this is the first study to measure and compare different types of secondary hematological malignancies post-breast cancer according to different postoperative treatment modalities. We were also able to answer questions involving rare outcomes with very bad prognostic features, which are of direct interest to patients, practicing oncologists, and cancer researchers.

Finally, the risk of these hematologic malignancies secondary to BC treatment is dynamic and will hopefully decrease once precision medicine and molecular subtyping and targeting become the standard of care. In fact, the recent dissemination in the routine setting of various prognostic gene signatures might help to reduce the number of patients exposed to cytotoxic chemotherapy in early-stage BC [42,43]. In a similar way, the increased risk related to radiation therapy should add another dimension to the question of whether women might choose a total mastectomy opposed to lumpectomy. It also raises the question of whether a shorter course of radiation should be preferred to traditional techniques. 

## 5. Conclusions

In conclusion, our population-based survey revealed increased risks of various hematological malignancies associated with postoperative early-stage BC treatment in the modern era. We found that long-term survivors of BC who received chemotherapy at an early age are most susceptible to developing AML, MDS, and ALL/LL. Patients who received radiation therapy were more prone to develop AML and HL/NHL. These results have important implications for the management of BC, especially in younger patients. Because the absolute benefit is related to risk, in low-risk for recurrence patients, the added benefit of chemotherapy and radiation therapy should take into consideration these long-term complications. 

## Figures and Tables

**Table 1:** Characteristics of the study population.

Variable	Overall	Surgery Only	Surgery and Radiotherapy	Surgery and Chemotherapy	All 3 Modalities
	324 056 (100)	50 323 (15.5)	151 360 (46.7)	13 937 (4.3)	108 436 (33.5)
**Age at cohort entry**					
Median age (IQR)	59 (50–68)	60 (50–70)	63 (53–71)	56 (47–64)	55 (47–64)
**Year of inclusion**					
2007–2009	105 560 (32.6)	20 768 (41.3)	44 251 (29.2)	7 057 (50.6)	33 484 (30.9)
2010–2012	109 120 (33.7)	14 862 (29.5)	52 284 (34.5)	3 784 (27.1)	38 190 (35.2)
2013–2015	109 376 (33.7)	14 693 (29.2)	54 825 (36.2)	3 096 (22.2)	36 762 (33.9)
**Affiliation to Complementary Universal Health Insurance ^a^**	14 257 (4.4)	2 211 (4.4)	4 862 (3.2)	861 (6.2)	6 323 (5.8)
**Type of structure ^b^**					
Public hospitals	110 282 (34.0)	18 462 (36.7)	49 294 (32.6)	5 095 (36.6)	37 431 (34.5)
Private hospitals	142 515 (44.0)	20 372 (40.5)	69 677 (46.0)	5 524 (39.6)	46 942 (43.3)
Cancer Centers	71 259 (22.0)	11 489 (22.8)	32 389 (21.4)	3 318 (23.8)	24 063 (22.2)
**Comorbidities ^c^**					
Severe alcoholism	4 902 (1.5)	790 (1.6)	2 300 (1.5)	212 (1.5)	1 600 (1.5)
Heavy smokers	19 178 (5.9)	3 184 (6.3)	8 838 (5.8)	782 (5.6)	6 374 (5.9)
Morbid obesity	40 548 (12.5)	6 178 (12.3)	20 047 (13.2)	1 547 (11.1)	12 776 (11.8)
Hypertension	114 488 (35.3)	18 072 (35.9)	60 469 (40.0)	4 062 (29.1)	31 885 (29.4)
Diabetes	32 277 (10.0)	4 996 (9.9)	16 059 (10.6)	1 315 (9.4)	9 907 (9.1)
Hyperlipidemia	69 146 (21.3)	10 530 (20.9)	37 937 (25.1)	2 331 (16.7)	18 348 (16.9)
Heart disease	37 814 (11.7)	7 019 (13.9)	20 895 (13.8)	1 309 (9.4)	8 591 (7.9)
HIV	436 (0.1)	87 (0.2)	168 (0.1)	26 (0.2)	155 (0.1)
Hepatitis B & C	1 536 (0.5)	263 (0.5)	764 (0.5)	59 (0.4)	450 (0.4)
**Comedications ^c^**					
Immunosuppressant	2 059 (0.6)	303 (0.6)	974 (0.6)	84 (0.6)	698 (0.6)
Antidepressants	47 725 (14.7)	7 769 (15.4)	23 099 (15.3)	1 897 (13.6)	14 960 (13.8)
Benzodiazepine	99 618 (30.7)	16 513 (32.8)	49 068 (32.4)	4 035 (29.0)	30 002 (27.7)
Contraception	36 566 (11.3)	5 005 (9.9)	12 783 (8.4)	1 610 (11.6)	17 168 (15.8)
Hormone replacement therapy	40 592 (12.5)	6 343 (12.6)	22 490 (14.9)	1 323 (9.5)	10 436 (9.6)
Breast cancer treatment*					
**Type of surgery**					
Partial mastectomy and lumpectomy	264 972 (81.8)	33 042 (65.6)	141 165 (93.3)	2 677 (19.2)	57 006 (76.3)
Radical mastectomy	59 084 (18.2)	17 287 (34.4)	10 195 (6.7)	11 260 (80.8)	25 715 (23.7)
**Hormonal therapy ^d^**	225 227 (69.5)	21 614 (43.0)	113 025 (74.7)	9 145 (65.6)	81 443 (75.1)
Aromatase Inhibitors	156 655 (48.3)	16 064 (31.9)	85 984 (56.8)	5 641 (40.5)	48 966 (45.2)
SERM	76 110 (23.5)	6 489 (12.9)	31 980 (21.1)	3 715 (26.7)	33 926 (31.3)
LHRH / GnRH agonist	2 769 (0.9)	220 (0.4)	938 (0.6)	204 (1.5)	1 407 (1.3)

Abbreviations: AI. Aromatase inhibitors; SERM. Selective estrogen receptor modulator; LHRH/GNRH agonist: Luteinizing hormone-releasing hormone/gonadotropin-releasing hormone; * Defined after cohort entry. ^a^ Free access to healthcare for people with an annual income less than 50% of the poverty threshold. ^b^ Structure where the surgery procedure for BC was performed. ^c^ Defined based on International Statistical Classification of Diseases and Related Health Problems Tenth Revision codes before cohort entry. ^d^ For any corresponding reimbursement registered within one year of cohort entry.

**Table 2:** Incidence of hematologic malignancies according to exposure group.

Hematologic Malignancy Type (*n* = 2 236)	Overall (1,647,704 PY)			Surgery Only (282,957 PY)			Surgery and Radiotherapy (746,236 PY)			Surgery and Chemotherapy (78,592 PY)			All 3 Modalities (540,311 PY)		
	No.	IR per 100 000 PY (95%CI)	Median Time from BC to HM (IQR)	No.	IR per 100 000 PY (95%CI)	Median Time from BC to HM (IQR)	No.	IR per 100 000 PY (95%CI)	Median Time from BC to HM (IQR)	No.	IR per 100 000 PY (95%CI)	Median Time from BC to HM (IQR)	No.	IR per 100 000 PY (95%CI)	Median Time from BC to HM (IQR)
**Myeloid Neoplasm**															
Acute myeloid leukemia	436	26.5 (24.0–29.0)	3.2 (2.1–5.0)	40	14.1 (10.1–19.2)	5.5 (2.8–7.5)	167	22.4 (19.1–26.0)	3.5 (2.3–5.1)	20	25.5 (15.5–39.3)	2.8 (2.4–3.6)	209	38.7 (33.6–44.3)	2.7 (1.8–4.2)
Myelodysplastic syndrome	552	33.5 (30.8–36.4)	4.0 (2.6–5.9)	98	34.6 (28.1–42.2)	4.2 (3.0–6.3)	272	36.5 (32.3–41.1)	3.9 (2.5–5.7)	29	36.9 (24.7–53.0)	4.1 (2.7–6.5)	153	28.3 (24.0–33.2)	3.9 (2.5–6.0)
Myeloproliferative Neoplasms	194	11.8 (10.2–13.5)	4.1 (2.6–6.8)	40	14.1 (10.1–19.3)	4.6 (2.1–6.9)	87	11.7 (9.3–14.4)	3.7 (2.7–6.0)	7	8.9 (3.6–18.3)	6.9 (3.5–7.6)	60	11.1 (8.5–14.3)	4.3 (2.8–7.0)
**Lymphoid Neoplasm**															
Multiple Myeloma	310	18.8 (16.8–21.0)	4.4 (2.9–6.5)	53	18.7 (14.0–24.5)	4.8 (3.4–6.7)	165	22.1 (18.9–25.8)	4.7 (3.2–6.5)	15	19.1 (10.7–31.5)	4.6 (2.8–7.6)	77	14.3 (11.2–17.8)	3.5 (2.5–5.6)
Hodgkin’s/Non-Hodgkin’s Lymphoma	668	40.6 (37.5–43.8)	4.7 (3.0–6.5)	111	39.3 (32.3–47.3)	4.5 (2.8–6.7)	382	51.2 (46.2–56.6)	4.6 (2.8–6.4)	26	33.1 (21.6–48.5)	6.4 (4.0–8.1)	149	27.6 (23.3–32.4)	4.8 (3.2–6.3)
Acute lymphoblastic leukemia/lymphocytic lymphoma	76	4.6 (3.6–5.8)	3.5 (2.2–5.7)	12	4.4 (2.3–7.7)	4.3 (2.5–6.2)	28	3.7 (2.5–5.4)	3.6 (2.3–5.1)	5	7.1 (2.3–16.5)	3.7 (1.8–5.6)	31	5.7 (3.8–8.0)	3.4 (2.0–6.1)

Abbreviations: No. Number of cases; PY. Person years; IR. Incidence Rate; BC. Breast Cancer; HM. Hematologic Malignancies; CI. Confidence Interval; IQR. Interquartile range.

**Table 3:** Risk of hematologic malignancies (HRs) by exposure groups.

Hematologic Malignancy Type (*n* = 2236)	Surgery (*n* = 50 321)	Surgery and Radiotherapy (*n* = 151 362)			Surgery and Chemotherapy (*n* = 13933)			All 3 Modalities (*n* = 108 440)		
		Age-aHR^1^ (95%)	aHR^2^ (95%)	*p*-value	Age-aHR^1^ (95%)	aHR^2^ (95%)	*p*-value	Age-aHR^1^ (95%)	aHR^2^ (95%)	*p*-value
**Myeloid Neoplasm**										
Acute myeloid leukemia	ref.	1.4 (1.0–2.0)	1.5 (1.0–2.1)	0.04	2.2 (1.3–3.8)	2.1 (1.2–3.6)	0.007	3.4 (2.4–4.8)	3.3 (2.3–4.7)	<0.0001
Myelodysplastic syndrome	ref.	1.0 (0.8–1.2)	1.0 (0.8–1.2)	0.8	1.7 (1.1–2.6)	1.7 (1.1–2.5)	0.02	1.4 (1.1–1.8)	1.4 (1.1–1.8)	0.01
Myeloproliferative Neoplasms	ref.	0.8 (0.5–1.1)	0.9 (0.6–1.3)	0.5	0.8 (0.4–1.8)	0.8 (0.4–1.9)	0.7	1.1 (0.7–1.7)	1.2 (0.8–1.8)	0.4
**Lymphoid Neoplasm**										
Multiple Myeloma	ref.	1.1 (0.8–1.5)	1.2 (0.9–1.6)	0.3	1.3 (0.8–2.4)	1.3 (0.7–2.4)	0.3	1.1 (0.8–1.5)	1.1 (0.8–1.6)	0.5
Hodgkin’s/Non-Hodgkin’s Lymphoma	ref.	1.3 (1.0–1.6)	1.3 (1.0–1.6)	0.02	1.1 (0.7–1.7)	1.1 (0.7–1.8)	0.5	1.0 (0.8–1.3)	1.0 (0.8–1.3)	0.8
Acute lymphoblastic leukemia/lymphocytic lymphoma	ref.	0.8 (0.4–1.6)	0.8 (0.4–1.7)	0.6	1.7 (0.6–4.8)	1.8 (0.6–5.3)	0.3	1.4 (0.7–2.7)	1.4 (0.7–2.8)	0.4

Abbreviations: CI. Confidence Interval; ref. Reference group. ^1^Age-aHR: Age adjusted Hazard Ratio; ^2^aHR: Adjusted Hazard Ratio. Multivariable Cox model adjusted for baseline characteristics including age at inclusion. Affiliation to complementary Universal health insurance. year of inclusion. type of structure. hormonal therapy. severe alcoholism. heavy smokers. morbid obesity. immunosuppressant. hepatitis B and C. HIV.

**Table 4:** Risk of hematologic malignancies (HRs) in patients exposed to chemotherapy and radiotherapy.

Hematologic Malignancy Type (*n* = 2236)	Chemotherapy (vs. no)			Radiotherapy (vs. no)		
	Age-aHR (95%)	aHR^a^ (95%)	*p*-value	Age-aHR (95%)	aHR^a^ (95%)	*p*-value
**Myeloid Neoplasm**						
Acute myeloid leukemia	2.4 (2.0–2.9)	2.3 (1.8–2.8)	<0.0001	1.7 (1.3–2.3)	1.5 (1.1–2.0)	0.005
Myelodysplastic syndrome	1.5 (1.2–1.8)	1.5 (1.2–1.8)	<0.0001	1.0 (0.8–1.2)	0.9 (0.8–1.2)	0.6
Myeloproliferative Neoplasms	1.3 (0.9–1.7)	1.3 (0.9–1.7)	0.1	0.9 (0.7–1.3)	1.0 (0.7–1.4)	0.8
**Lymphoid Neoplasm**						
Multiple Myeloma	1.0 (0.8–1.3)	1.0 (0.8–1.3)	0.9	1.1 (0.8–1.4)	1.1 (0.8–1.4)	0.5
Hodgkin’s/Non-Hodgkin’s Lymphoma	0.9 (0.7–1.0)	0.8 (0.7–1.1)	0.06	1.2 (1.0–1.4)	1.2 (1.0–1.5)	0.05
Acute lymphoblastic leukemia/lymphocytic lymphoma	1.6 (1.0–2.6)	1.7 (1.0–2.7)	0.03	0.9 (0.5–1.6)	0.8 (0.5–1.4)	0.5

Age-aHR: Age adjusted Hazard Ratio; aHR: Adjusted Hazard Ratio. ^a^ Multivariable Cox model adjusted for baseline characteristics including age at inclusion. affiliation to complementary Universal health insurance. Year of inclusion. type of structure. radiotherapy. chemotherapy. severe alcoholism. heavy smokers. morbid obesity. immunosuppressant. hepatitis B and C. HIV

## Supplementary Materials

The following are available online at https://www.mdpi.com/2072-6694/11/10/1463/s1, Figure S1: Cumulative Incidence function of hematologic malignancies by Exposure groups, Figure S2: Survival curves after hematologic malignancies, Table S1: Detailed definition of the exposure with the corresponding codes, Table S2: Detailed definition of the covariates with the corresponding codes, Table S3: Detailed definition of the outcome subtypes with their ICD-10 codes, Table S4: Associations between exposure groups, covariates, and the risk of hematologic malignancies in the full Cox Multivariable model, Table S5: Incidence and Risk of hematologic malignancies (HRs) in patients exposed to hormone therapy, Table S6: Fine and Gray analyses: Sensitivity analyses.
