# Supplementary material for: Risk of Hematologic Malignant Neoplasms after Postoperative Treatment of Breast Cancer
Source: Cancers (Basel). 2019 Sep 29;11(10):1463. doi: 10.3390/cancers11101463 (PMC6827362; doi:10.3390/cancers11101463)
Supplement: Supplementary file 1 [file cancers-11-01463-s001.pdf]

Supplementary Materials

Risk of Hematologic Malignant Neoplasms after Postoperative Treatment of Breast Cancer

Supplementary Tables

Table S1. Detailed definition of the exposure with the corresponding codes.

Variable	International Classification of Diseases, 10th Revision (ICD-10) Codes	Anatomical Therapeutic Chemical (ATC) Classification System Code	French Medical Common Procedure Coding System	Delay Used for Algorithm Construction
**Surgery**	–	–	QEFA019 QEFA017 QEFA008 QEFA003 QEFA007 QEFA005 QEFA010 QEFA020 QEFA015 QEFA013 QEFA012 QEFA016 QEFA001 QEFA004	In the 6 months after breast cancer diagnosis
**Radiation Therapy**	Z5101	–	ZZNL040 ZZNL042 ZZNL039 ZZNL037 ZZNL034 ZZNL036 ZZNL043 ZZNL045 ZZNL049 ZZNL050 ZZNL048 ZZNL047 ZZNL046 ZZNL033 ZZNL031 ZZNL020 ZZNL021 ZZNL016 ZANL001 AANL001 ZZNL023 ZZNL024 ZZNL028 ZZNL030 ZZNL027 ZZNL026 ZZNL025 ZZNL051 ZZNA002 ZZNL052 ZZNL903 KCNL004 PANL001 ZZNL902 ZZNL904 FENL001 AANL002 ZZNL906 ZZNL905 AZNL001 EZNF900 QZNL001 KCNL003 ZZNL055 ZZNL058 ZZNL900 ZZNL053 ZZNL054 ZZNL060 ZZNL059 ZZNL061 ZZNL066 ZZNL064 ZZNL065 ZZNL063 ZZNL062 ZZMK003 ZZMK004 ZZMK002 ZZMK001 ZZMK005 ZZMK013 ZZMK012 ZZMK011 ZZMK010 ZZMK007 ZZMK006 YYYY493 YYYY511 YYYY520 YYYY500 YYYY497 ZZMK014 YYYY522 YYYY533 YYYY577 YYYY588 YYYY566 YYYY555 YYYY544 YYYY599 ZZMK028 ZZMP008 ZZMP010 ZZMP001 ZZML003 ZZML002 ZZMP011 ZZMP012 ZZMP900 YYYY492 ZZMP017 ZZMP016 ZZMP013 ZZML001 ZZMK900 ZZMK019 ZZMK020 ZZMK018 ZZMK017 ZZMK016 ZZMK021 ZZMK022 ZZMK026 ZZMK027 ZZMK025 ZZMK024 ZZMK023 ZZMK015 YYYY470 YYYY299 YYYY267 YYYY301 YYYY302 YYYY303 YYYY256 YYYY244 YYYY175 YYYY197 YYYY211 YYYY223 YYYY304 YYYY305 YYYY316 YYYY315 YYYY320 YYYY323 YYYY324 YYYY314 YYYY313 YYYY306 YYYY307 YYYY310 YYYY312 YYYY152 YYYY141 YYYY045 YYYY023 YYYY046 YYYY047 YYYY048 YYYY021 QZMP003 AGMP001 AZMP001 HPLA002 JDFA014 YYYY049 YYYY050 YYYY099 YYYY101 YYYY122 YYYY136 YYYY056 YYYY055 YYYY051 YYYY052 YYYY053 YYYY054 YYYY325 YYYY326 YYYY391 YYYY390 YYYY392 YYYY393 YYYY398 YYYY387 YYYY383 YYYY379 YYYY380 YYYY381 YYYY458 YYYY459 YYYY460 YYYY377 YYYY371 YYYY343 YYYY338 YYYY345 YYYY346 YYYY382 YYYY450 YYYY451 YYYY471 YYYY479 YYYY480 YYYY481 YYYY469 YYYY468 YYYY457 YYYY347 YYYY337	In the year following inclusion

**Chemotherapy**	Z511	–	YYYY336 YYYY327 YYYY331 YYYY334 YYYY335 YYYY348 YYYY349 YYYY367 YYYY368 YYYY369 YYYY370 YYYY365 YYYY360 YYYY356 YYYY357 YYYY358 YYYY359 YYYY491 HPLB007 HPLB002 GGLB001 HPLB003 ZZLF004 AFLB003 GGLB008 AFLB013 EDLF019 EBLF003 ECLF006 EELF004 EDLF020 EDLF015 EDLF014 EDLF018 EDLF021 EELF005 EBLF002 ECLF005 EDLF017 EDLF016 ABLB006 ZZLF900	In the 6 months following inclusion
**Hormonotherapy**		L02BA01 L02BA02 L02BA03 L02BG03 L02BG06 L02BG04 L02AE03 L02AE02	–	Any reimbursement happening in the year after inclusion

Table S2. Detailed definition of the covariates with the corresponding codes.

Covariates	International Classification of Diseases, 10th Revision (ICD–10) Codes	Anatomical Therapeutic Chemical (ATC) Classification System Code	French Medical Common Procedure Coding System	Delay Used for Algorithm Construction
**Measurable History of Smoking**	F17 Z71.6 Z72.0 I731 J41 J42 J43 J44 T65.2	N06AX12 N07BA		One year before inclusion and at inclusion
**Morbid Obesity**	Z68.3 Z68.4 E65 E66	A08AB01, A08AX01, A10B	HFCA001 HFCC003 HFFA001 HFFA011 HFFC004 HFFC018 HFMA009 HFMC008 HFMA011 HFKA001 HFMA010 HFMC006 HFMC007 HGCA009 HGCC027 HFLE002 HFLC900 HFGC900 HFKC001 HFKA002	Two years before inclusion and at inclusion
**Alcohol Use Disorder**	F10 K70 T51 E24.4 G31.2 G62.1 G72.1 I42.6 K29.2 K86.0 T51.0 T51.9 Z50.2 Z71.4 Z72.1	N07BB01, N07BB02, N07BB03, N07BB04, N07BB05		One year before inclusion and at inclusion
**Contraception (Oral Contraceptive Pill / Implant, Transdermal Patch, Intrauterine Device)**	1101938, 1103848, 1106752, 1111760, 1120717, 1121125, 1122283, 1125749, 1128370, 1132519, 1132531, 1134760, 1135890, 1146770, 1152960, 1158536, 1167363, 1171407, 1173062, 1187615	G02BB01, G03AA, G03AB, G03AC, G02BA01, G02BA02, G02BA03	JKLD001, JKGA001, JKGC001, JKGD001, JKGD004, JKGE001, JKKD001, QZGA001, QZGA002, QZGA003, QZGA004, QZGA006, QZGA007, QZGA008, QZGA010	Any reimbursement registered 6 months before cohort entry and 5 years for intrauterine device
**Hormone Replacement Therapy**		G03XC G03CA G03CC G03CX G03CA G03CC G03CX G03FA G03FB G03FA G03FB G03HB G03CA G03CC G03CX G03FA G03FB G03HB		Any reimbursement registered 6 months before cohort entry

Table S3. Detailed definition of the outcome subtypes with their ICD–10 codes.

Hematological Malignancies		Subtypes (ICD-10 Codes)
**Myeloid Neoplasm**	Acute myeloid leukemia (AML)	Acute myeloid leukemia **(C92.0/C92.8/C92.4/C92.5/C92.6)**
		Acute myeloid promyelocytic leukemia **(C92.4)**
	Myelodysplastic Syndrome (MDS)	Myelodysplastic Syndrome **(D46)**
	Myeloproliferative neoplasm (MPN)	Chronic myeloid leukemia **(C92.1)**
		Chronic myeloproliferative disease **(D47.1)**
**Lymphoid Neoplasm**	Multiple Myeloma (MM)	Multiple myeloma and malignant plasma cell neoplasms **(C90)**
	Hodgkin's and Non-Hodgkin's lymphoma (HL/NHL)	Hodgkin lymphoma **(C81)**
		Chronic lymphocytic leukemia of B-cell type **(C91.1)**
		Small cell B-cell lymphoma **(C83.0)**
		Follicular lymphoma **(C82)**
		Diffuse large B-cell lymphoma **(C83.3)**
		Mantle cell lymphoma **(C83.1)**
		MALT-lymphoma **(C88.4)**
		Other specified types of T/NK–cell lymphoma **(C86)**
		Other nonfollicular lymphoma **(C83.8)**
		Waldenström macroglobulinemia **(C88.0)**
		Neoplasm of uncertain behavior of lymphoid and related tissue **(D47.9)**
	Acute Lymphoblastic Leukemia /Lymphoblastic Lymphoma (ALL/LL)	Acute lymphoblastic leukemia **(C91.0)**
		Adult T-cell lymphoma/leukemia **(C91.5)**
		Lymphoblastic (diffuse) lymphoma **(C83.5)**

Table S4. Associations between exposure group covariates and the risk of hematologic malignancies in the full Cox Multivariable model.

	AML		MDS		MPN		MM		HL/NHL		ALL/LL	
	**aHR (95%CI)**	***p* value**	**aHR (95%CI)**	***p* value**	**aHR (95%CI)**	***p* value**	**aHR (95%CI)**	***p* value**	**aHR (95%CI)**	***p* value**	**aHR (95%CI)**	***p* value**
**Age at cohort entry** *(per 1 year increase)*	1.0 (1.0–1.1)	<.0001	1.1 (1.1–1.1)	<.0001	1.1 (1.0–1.1)	<.0001	1.1 (1.0–1.1)	<.0001	1.1 (1.0–1.1)	<.0001	1.0 (1.0–1.0)	0.4
**Main Exposure Groups**												
Surgery												
Surgery and Radiotherapy	1.5 (1.0–2.1)	0.04	1.0 (0.8–1.2)	0.8	0.9 (0.6–1.3)	0.5	1.2 (0.9–1.6)	0.3	1.3 (1.0–1.6)	0.02	0.8 (0.4–1.7)	0.6
Surgery and Chemotherapy	2.1 (1.2–3.6)	0.007	1.7 (1.1–2.5)	0.02	0.8 (0.4–1.9)	0.7	1.3 (0.7–2.4)	0.3	1.1 (0.7–1.8)	0.5	1.8 (0.6–5.3)	0.3
All 3 modalities	3.3 (2.3–4.7)	<.0001	1.4 (1.1–1.8)	0.01	1.2 (0.8–1.8)	0.40	1.1 (0.8–1.6)	0.5	1.0 (0.8–1.3)	0.8	1.4 (0.7–2.8)	0.4
Hormone therapy	1.2 (0.9–1.5)	0.10	1.2 (1.0–1.5)	0.07	1.0 (0.6–1.6)	0.10	1.0 (0.8–1.3)	0.8	1.1 (0.8–1.4)	0.4	1.0 (0.7–1.3)	0.9
**Comorbidities**												
Hepatitis B & C	0.0 (0.0–*)	1.0	1.1 (0.4–3.6)	0.8	0.0 (0.0–.)	1.0	1.5 (0.4–6.1)	0.6	1.8 (0.8–4.4)	0.2	4.1 (0.6–29.7)	0.2
HIV	0.0 (0.0–*)	1.0	2.8 (0.4–20.3)	0.3	0.0 (0.0–.)	1.0	4.0 (0.5–29.8)	0.2	0.0 (0.0–*)	1.0	0.0 (0.0–*)	1.0
Severe alcoholism	0.4 (0.1–1.7)	0.2	1.3 (0.7–2.6)	0.4	0.9 (0.2–3.6)	0.8	0.9 (0.3–3.0)	0.9	1.1 (0.5–2.2)	0.8	0.0 (0.0–*)	1.0
Immunosuppressants	0.5 (0.1–3.7)	0.5	2.6 (1.1–5.7)	0.02	1.1 (0.2–8.0)	0.9	0.7 (0.1–5.0)	0.7	0.9 (0.3–2.8)	0.9	0.0 (0.0–*)	1.0
Heavy smokers	0.9 (0.5–1.5)	0.7	1.0 (0.7–1.5)	0.9	1.1 (0.6–2.1)	0.8	1.2 (0.7–2.0)	0.5	1.1 (0.7–1.5)	0.8	0.3 (0.0–2.2)	0.2
Morbid obesity	1.0 (0.6–1.7)	0.9	0.8 (0.5–1.2)	0.2	1.7 (0.9–3.1)	0.1	1.8 (1.1–2.9)	0.02	0.9 (0.6–1.3)	0.6	0.4 (0.1–1.4)	0.1
**CMU**	0.6 (0.3–1.3)	0.2	1.6 (1.0–2.6)	0.07	1.4 (0.6–3.1)	0.4	1.7 (0.9–3.2)	0.09	1.1 (0.6–1.8)	0.8	1.3 (0.4–4.3)	0.7
**Region**	NS	NS	NS	NS	NS	NS	NS	NS	NS	NS	NS	NS
**Year of Inclusion**												
2007	ref	ref	ref	ref	ref	ref	ref	ref	ref	ref	ref	ref
2008	0.8 (0.5–1.1)	1.1	1.0 (0.7–1.3)	0.9	0.6 (0.4–1.0)	0.07	1.0 (0.7–1.5)	0.9	1.2 (0.9–1.6)	0.2	1.6 (0.6–4.1)	0.4
2009	0.7 (0.5–1.1)	1.1	0.8 (0.6–1.1)	0.2	0.8 (0.5–1.3)	0.4	0.9 (0.6–1.4)	0.7	0.9 (0.6–1.2)	0.4	2.2 (0.8–5.8)	0.1
2010	0.8 (0.5–1.1)	1.1	0.7 (0.5–1.1)	0.1	0.8 (0.5–1.4)	0.4	0.7 (0.4–1.1)	0.1	1.2 (0.9–1.7)	0.2	1.8 (0.6–5.1)	0.3
2011	0.7 (0.4–1.0)	1.0	0.9 (0.6–1.3)	0.6	0.6 (0.3–1.2)	0.1	0.5 (0.3–0.9)	0.01	1.0 (0.7–1.4)	0.9	2.9 (1.1–7.9)	0.0
2012	0.7 (0.5–1.1)	1.1	0.8 (0.6–1.2)	0.4	0.9 (0.5–1.7)	0.8	0.8 (0.5–1.4)	0.5	1.1 (0.8–1.6)	0.5	0.9 (0.2–3.6)	0.9
2013	0.7 (0.5–1.1)	1.1	0.5 (0.3–0.8)	0.003	0.6 (0.3–1.2)	0.2	1.0 (0.6–1.7)	0.9	1.3 (0.9–1.9)	0.1	1.4 (0.4–5.0)	0.6
2014	0.4 (0.2–0.7)	0.7	0.9 (0.6–1.4)	0.6	0.1 (0.0–0.6)	0.02	0.8 (0.4–1.5)	0.5	1.3 (0.9–2.0)	0.2	1.9 (0.5–7.1)	0.3
2015	0.6 (0.3–1.2)	1.2	0.7 (0.3–1.3)	0.2	0.5 (0.1–1.7)	0.3	0.3 (0.1–1.1)	0.07	0.8 (0.4–1.6)	0.6	1.6 (0.3–8.2)	0.6
**Type of structure**												
Public hospitals	ref	ref	ref	ref	ref	ref	ref	ref	ref	ref	ref	ref
Private hospitals	1.0 (0.7–1.3)	0.8	1.0 (0.7–1.2)	0.8	1.2 (0.8–1.8)	0.5	1.4 (1.0–1.9)	0.08	0.8 (0.6–1.0)	0.07	0.9 (0.4–1.8)	0.7
Cancer Centers	0.7 (0.5–0.9)	0.001	0.8 (0.6–1.0)	0.03	0.7 (0.5–1.0)	0.05	1.0 (0.7–1.3)	0.9	0.9 (0.8–1.1)	0.4	0.8 (0.5–1.5)	0.5

Table S5. Incidence and Risk of hematologic malignancies (HRs) in patients exposed to hormone therapy.

	**Hormone Therapy (yes vs. no) (*n* = 225 227)**					
**Hematologic Malignancy Type (*n* = 1 623)**	**No.**	**IR per 100 000 PY (95%CI)**	**Median Time from BC to HM (IQR)**	**Age–aHR (95%)**	**aHR (95%CI)**	***p*–value**
**Myeloid Neoplasm**						
Acute myeloid leukemia	332	29,2 (26,2–32,5)	3,2 (2,1–4,9)	1,3 (1,1–1,7)	1,2 (0,9–1,5)	0,1
Myelodysplastic syndrome	420	37,0 (33,5–40,7)	3,9 (2,6–5,8)	1,2 (1,0–1,5)	1,2 (1,0–1,5)	0,07
Myeloproliferative Neoplasms	128	11,3 (9,4–13,4)	4,0 (2,6–6,8)	0,8 (0,6–1,1)	1,0 (0,6–1,6)	0,1
**Lymphoid Neoplasm**						
Multiple Myeloma	220	19,4 (16,9–22,1)	4,3 (2,8–6,3)	1,0 (0,8–1,3)	1,0 (0,8–1,3)	0,8
Hodgkin/Non Hodgkin Lymphoma	471	41,5 (37,8–45,4)	4,8 (3,0–6,7)	1,0 (0,8–1,1)	1,1 (0,8–1,4)	0,4
Acute lymphoblastic leukemia/lymphocytic lymphoma	52	4,6 (3,4–6,0)	3,5 (2,0–5,8)	1,0 (0,6–1,6)	1,0 (0,7–1,3)	0,9

Abbreviations: No. number of cases; PY. Person years; IR. Incidence Rate; BC. Breast Cancer; HM. Hematologic Malignancies; CI. Confidence Interval; IQR. Interquartile range; [1]Age–aHR: Age adjusted Hazard Ratio; [2]aHR: Adjusted Hazard Ratio. Multivariable Cox model adjusted for baseline characteristics including age at inclusion. affiliation to complementary Universal health insurance. Year of inclusion. type of structure. radiotherapy. chemotherapy. severe alcoholism. heavy smokers. morbid obesity. immunosuppressant. hepatitis B & C. HIV.

Table S6. Risk of hematologic malignancies (HRs) by exposure groups (Fine and Gray).

Hematologic Malignancy Type (*n* = 2236)	Surgery (*n* = 50 321)	Surgery and Radiotherapy (*n* = 151 362)	Surgery and Chemotherapy (*n* = 13 933)	All 3 Modalities (*n* = 108 440)	Surgery (*n* = 50 321)	Surgery and Radiotherapy (*n* = 151 362)	Surgery and Chemotherapy (*n* = 13 933)	All 3 Modalities (*n* = 108 440)
	Age–HR[a] (95%)				aHR[b] (95%)			
**Myeloid Neoplasm**								
Acute myeloid leukemia	ref.	1.5 (1.0–2.1)	2.1 (1.2–3.6)	3.3 (2.3–4.6)	ref.	1.5 (1.0–2.1)	2.0 (1.2–3.4)	3.2 (2.3–4.6)
Myelodysplastic syndrome	ref.	1.0 (0.8–1.2)	1.6 (1.0–2.4)	1.4 (1.0–1.7)	ref.	1.0 (0.8–1.3)	1.5 (1.0–2.3)	1.3 (1.0–1.7)
Myeloproliferative Neoplasms	ref.	0.8 (0.5–1.2)	0.8 (0.3–1.7)	1.0 (0.7–1.6)	ref.	0.9 (0.6–1.3)	0.8 (0.3–1.7)	1.1 (0.7–1.7)
**Lymphoid Neoplasm**								
Multiple Myeloma	ref.	1.1 (0.8–1.6)	1.3 (0.7–2.2)	1.0 (0.7–1.5)	ref.	1.2 (0.9–1.6)	1.2 (0.7–2.2)	1.1 (0.7–1.6)
Hodgkin/Non Hodgkin Lymphoma	ref.	1.3 (1.0–1.6)	1.0 (0.7–1.6)	1.0 (0.7–1.2)	ref.	1.3 (1.1–1.6)	1.0 (0.7–1.6)	1.0 (0.8–1.3)
Acute lymphoblastic leukemia/lymphocytic lymphoma	ref.	0.8 (0.4–1.6)	1.6 (0.6–4.6)	1.3 (0.7–2.6)	ref.	0.8 (0.4–1.7)	1.7 (0.6–4.9)	1.3 (0.7–2.6)

[a] age–HR: age Adjusted Hazard Ratio. [b]aHR: Adjusted Hazard Ratio in Multivariable Fine and Gray model adjusted for baseline characteristics including age at inclusion. affiliation to complementary Universal health insurance. Year of inclusion. type of structure. chemotherapy. hormonal therapy. severe alcoholism. heavy smokers. morbid obesity. immunosuppressant. hepatitis B & C. HIV.

Supplementary Figures

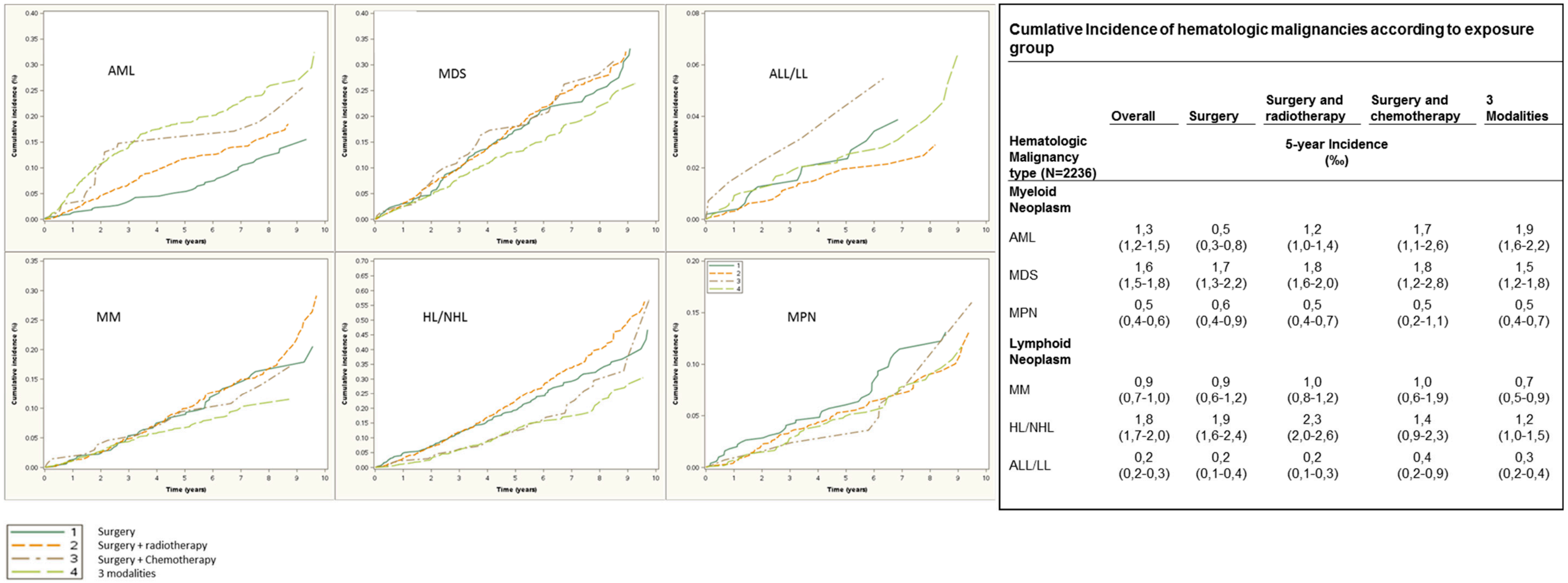

Cumlative Incidence of hematologic malignancies according to exposure group

Hematologic Malignancy type (N=2236)	Overall	Surgery	Surgery and radiotherapy	Surgery and chemotherapy	3 Modalities
	5-year Incidence (‰)				
**Myeloid Neoplasm**					
AML	1,3 (1,2-1,5)	0,5 (0,3-0,8)	1,2 (1,0-1,4)	1,7 (1,1-2,6)	1,9 (1,6-2,2)
MDS	1,6 (1,5-1,8)	1,7 (1,3-2,2)	1,8 (1,6-2,0)	1,8 (1,2-2,8)	1,5 (1,2-1,8)
MPN	0,5 (0,4-0,6)	0,6 (0,4-0,9)	0,5 (0,4-0,7)	0,5 (0,2-1,1)	0,5 (0,4-0,7)
**Lymphoid Neoplasm**					
MM	0,9 (0,7-1,0)	0,9 (0,6-1,2)	1,0 (0,8-1,2)	1,0 (0,6-1,9)	0,7 (0,5-0,9)
HL/NHL	1,8 (1,7-2,0)	1,9 (1,6-2,4)	2,3 (2,0-2,6)	1,4 (0,9-2,3)	1,2 (1,0-1,5)
ALL/LL	0,2 (0,2-0,3)	0,2 (0,1-0,4)	0,2 (0,1-0,3)	0,4 (0,2-0,9)	0,3 (0,2-0,4)

Figure S1. Cumulative Incidence function of hematologic malignancies by Exposure groups.

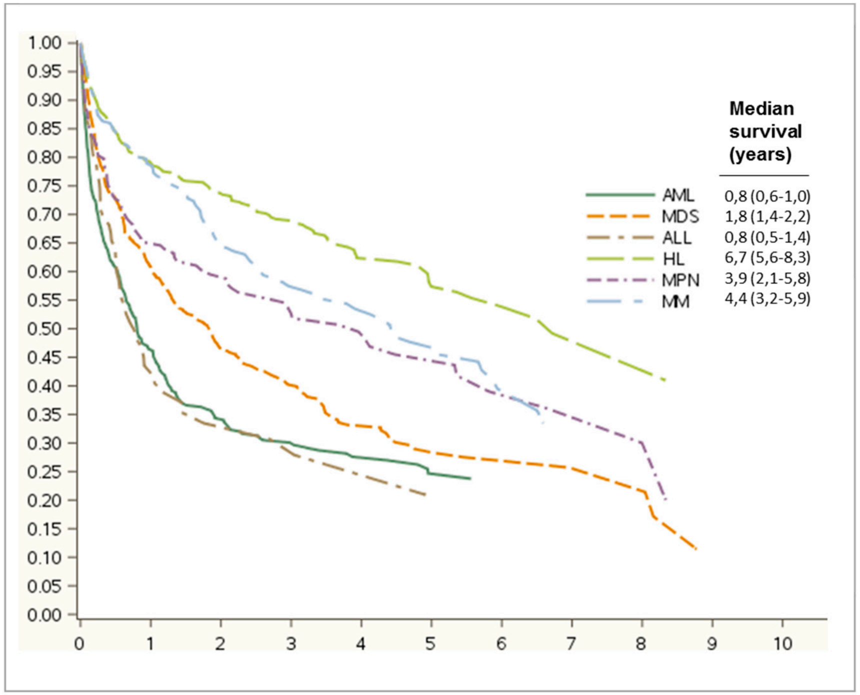

Figure S2. Survival curves after hematologic malignancie.
